# Ring-like late gadolinium enhancement: differential diagnosis and mimics

**DOI:** 10.1590/0100-3984.2024.0111

**Published:** 2025-02-27

**Authors:** André Vaz, Kevin Rafael De Paula Morales, Eduardo Kaiser Ururahy Nunes Fonseca, Juliana Pato Serra Souza, Maria Júlia Silveira Rahal, Ludmila Mintzu Young, Leticia Muniz Pereira, Luiz Raphael Pereira Donoso Scoppetta, José Rodrigues Parga Filho

**Affiliations:** 1 Instituto do Coração do Hospital das Clínicas da Faculdade de Medicina da Universidade de São Paulo (InCor/HC-FMUSP), São Paulo, SP, Brazil

**Keywords:** Cardiomyopathies, Arrhythmogenic right ventricular cardiomyopathy, Magnetic resonance imaging, Gadolinium, Cardiomiopatias, Cardiomiopatia ventricular direita arritmogêncica, Ressonância magnética, Gadolínio

## Abstract

Advances in cardiac magnetic resonance have promoted tissue characterization with high spatial and contrast resolution, and late gadolinium enhancement (LGE) sequences have improved the detection of myocardial fibrosis. The distribution pattern of LGE facilitates differentiation between ischemic and nonischemic etiologies and aids in refining diagnoses within nonischemic cardiomyopathies, suggesting specific etiological factors. A distinctive nonischemic LGE pattern that has recently gained prominence is the ring-like pattern, defined as a subepicardial or mid-wall circumferential or semi-circumferential enhancement, which involves at least three contiguous segments within the same short-axis slice. Initially identified as a diagnostic marker for desmoplakin and filamin C-related cardiomyopathies, the pattern has been reported in nongenetic conditions; nevertheless, it remains an uncommon finding in these diseases. In this article, we aim to present the differential diagnoses of ring-like LGE and its mimics. The combination of epidemiological, clinical, electrocardiographic, and additional features enables a focused refinement of the differential diagnosis associated with ring-like LGE.

## Abstract (pt)

Os avanços na ressonância magnética cardíaca possibilitaram a caracterização do tecido com alta resolução espacial e de contraste, ao passo que as sequências de realce tardio (RT) de gadolínio contribuíram para a melhoria da detecção da fibrose miocárdica. O padrão de distribuição do RT facilita a diferenciação entre etiologias isquêmicas e não isquêmicas e ajuda a refinar os diagnósticos dentro das cardiomiopatias não isquêmicas, sugerindo fatores etiológicos específicos. Um padrão distinto de RT não isquêmico que ganhou destaque recentemente é o padrão em forma de anel (*ring-like*), definido como um realce subepicárdico ou circunferencial ou semicircunferencial da parede média, que envolve pelo menos três segmentos contíguos de um mesmo corte no eixo curto. Inicialmente identificado como um marcador de diagnóstico para a desmoplaquina e a filamina C, o padrão foi relatado em condições não genéticas; contudo, continua sendo um achado incomum nessas doenças. Neste artigo, nosso objetivo é apresentar os diagnósticos diferenciais do RT em forma de anel e seus imitadores. A combinação de características epidemiológicas, clínicas, eletrocardiográficas e adicionais permite um refinamento do diagnóstico diferencial do RT em forma de anel.

## INTRODUCTION

Late gadolinium enhancement (LGE) on cardiovascular magnetic resonance (CMR) imaging showcases distinct patterns whose distribution promotes differentiation between ischemic and nonischemic etiologies. Specific LGE features can further refine the diagnosis within nonischemic cardiomyopathies^(1)^. Typically, LGE is classified as follows: subendocardial, transmural, mid-wall, subepicardial, junctional, or multifocal. Subendocardial and transmural enhancement suggest an ischemic etiology when following coronary territories, whereas the other patterns suggest a nonischemic etiology^(1)^.

A recent study highlighted a particular nonischemic pattern known as ring-like LGE, defined as involvement of the subepicardial or mid-wall layer in at least three contiguous left ventricle (LV) segments in the same short-axis section^(2)^. Originally, ring-like LGE was described in cardiomyopathies with desmoplakin and filamin-C gene variants, although it has also been documented in arrhythmogenic cardiomyopathy (ACM) and dilated cardiomyopathy (DCM) associated with other variants^(2^,^3^,^4^,^5)^. However, some inflammatory cardiomyopathies with extensive myocardial involvement may display a ring-like LGE and other diseases with a completely different clinical scenario may sporadically present a circumferential LGE, mimicking the ring-like pattern. It should be emphasized that ring-like LGE is not the typical presentation of these diseases, and other CMR findings, clinical history, and ancillary studies are essential to ensure the correct diagnosis. Therefore, the objective of this article is to present a systematic approach to the differential diagnoses of ring-like LGE, including a summary of clinical findings and complementary exams that aid in the diagnosis. Ring-like LGE mimics are also briefly addressed.

## GENETIC CAUSES OF RING-LIKE LGE

Genetic causes of ring-like LGE are associated with a family history of cardiomyopathy or premature sudden death. These diseases, which include ACM and idiopathic DCM, present with a hypokinetic nondilated LV or DCM phenotype, usually with no myocardial edema. Some cases exhibit genotypic and phenotypic overlap, and this particularity will be addressed. Table 1 summarizes the clinical, imaging, and ancillary findings that support a specific diagnosis of ring-like LGE.

**Table 1:** Summary of the clinical, imaging, and ancillary findings of causes of ring-like LGE.

Diagnosis	Clinical context	Electrocardiogram	Magnetic resonance imaging	Ancillary tests
ACM	Palpitations, syncope, and cardiac arrest	Low voltages in limb leads Epsilon waves Negative T waves Ventricular arrhythmias	Dilatation, systolic dysfunction, and regional wall motion abnormality of the RV or LV “Rat-bite” appearance in the LV	Fibrous replacement of the myocardium, with or without fatty tissue on endomyocardial biopsy
DCM	Heart failure	Normal Nonspecifc T-wave changes (left bundle branch block)	LV dilatation and systolic dysfunction Septal mid-wall fbrosis	Negative investigation for other underlying pathologies
Acute myocarditis	Flu-like or gastrointestinal prodromes Chest pain, dyspnea, and fever	Atrioventricular block PQ depression with ST-segment elevation QT-interval prolongation T-wave inversion	Myocardial edemaInferolateral LGE	Increased C-reactive protein and troponin Viral serology testing not recommended
Acute giant cell and eosinophilic myocarditis	Prior autoimmune disease, hypersensitivity, eosinophilic granulomatosis with polyangiitis, hypereosinophilic syndromes, and parasitic infection Fulminant myocarditis Rapidly progressive heart failure	Ventricular arrhythmias and atrioventricular block	Myocardial edema Subendocardial LGE Septal LGE	Eosinophilia
Heart transplant rejection	Heart transplantation Asymptomatic or nonspecifc, insidious symptoms in mild cases Hemodynamic compromise in fulminant cases	Electrical conduction abnormalities	Myocardial edema Septal LGE	Myocardial infammatory infltration on endomyocardial biopsy of acute cellular rejection CD68+ and C4d+ in acute antibody-mediated rejection
Cardiac sarcoidosis	Syncope Unexplained nonischemic heart failure in young adults	Atrioventricular block and ventricular arrhythmias	Myocardial edema Septal LGE LGE extending to the RV	Myocardial infammatory activity on FDG-PET Noncaseating epithelioid granulomas in endomyocardial biopsy

### ACM

**Definition** – ACM is an inherited disease characterized by fibrofatty infiltration of the myocardium that predisposes individuals, particularly young men, to potentially fatal arrhythmias^(6)^. It presents as a right-dominant, biventricular, or left-dominant phenotype^(7^,^8)^. According to the 2020 International Criteria (“Padua criteria”), the major CMR criteria for diagnosing right-dominant ACM are regional akinesia, dyskinesia, or bulging, accompanied by global dilatation or systolic dysfunction; and transmural LGE in one or more regions, detected in two orthogonal views^(6^,^7^,^9)^. Conversely, the major criterion for diagnosing left-dominant ACM is LGE in one or more segments, detected in two orthogonal views, excluding the septal or junctional pattern; in the LV, dilatation and dysfunction are considered minor criteria^(6^,^7^,^9)^. Recently, the European Task Force proposed an update to the diagnostic criteria for ACM, in which it included the ring-like LGE pattern as a major structural criterion. That update also downgraded to minor criteria LGE in the right ventricle (RV) and other LGE patterns in the LV^(10)^. Despite being one of the pathological hallmarks of ACM, myocardial fat deposits seen on CMR are not considered an accurate feature because of the uncertainty of reproducibility and the lack of a control population^(11)^. Biventricular and left-dominant ACM are characterized, respectively, by biventricular morphofunctional or structural criteria and by isolated LV structural criteria plus ACM gene-related variations^(6^,^7^,^9)^. Our discussion will focus on the biventricular and left-dominant ACM phenotypes because they can be associated with ring-like LGE^(2^,^3)^.

**Etiology** – In most cases, ACM is associated with variations in genes encoding components of intercellular junctions. These genes encode desmosomal and non-desmosomal proteins. The right-dominant phenotype is predominantly associated with variations in desmosomal genes, including plakophilin 2, junction plakoglobin, desmoglein 2, and desmocollin 2^(7)^. In contrast, biventricular and left-dominant ACM are usually associated with variations in nondesmosomal genes, specifically phospholamban, filamin-C, desmin, titin, lamin A/C (LMNA), and RNA binding motif protein 20. Of note, desmoplakin variations are the only desmosomal variation associated with primarily non-right-dominant phenotypes^(2^,^8^,^12^,^13^,^14)^.

**Epidemiology** – The prevalence of ACM, including that of the right-dominant phenotype, is estimated at 1:5,000 population^(6^,^12)^. It is considered a major cause of sudden death, particularly in athletes and young men^(6^,^12)^.

**Clinical manifestations** – Palpitations, syncope, and cardiac arrest are the primary symptoms reported in cases of ACM. A family history of premature sudden death (at < 35 years of age) or of an ACM diagnosis may also be present^(6^,^9^,^12^,^15)^. Electrocardiogram and 24-h Holter monitoring are essential to detect low voltages, epsilon waves, negative T waves, and a high burden of ventricular arrhythmias, defined as > 500 ventricular extrasystoles per 24 h, nonsustained and sustained ventricular tachycardia, especially with right bundle branch block morphology^(6^,^9^,^12^,^15)^.

Unique clinical features may be traced to specific gene variations. Phospholamban-, filamin-C-, and LMNA-related ACM present a high risk of sudden death^(2^,^7^,^12^,^13^,^14)^. Desmoplakin-related ACM may present with Carvajal syndrome^(7^,^12^,^13^,^14)^, which is a cardiocutaneous syndrome characterized by left-dominant ACM, woolly hair, and palmoplantar keratoderma, and “hot phases”, which have an acute clinical presentation simulating myocarditis or acute coronary syndrome^(7^,^12^,^13^,^14)^. Desmin-related and filamin-C-related ACM may be associated with myofibrillar myopathy, conduction disorders, and an overlap with hypertrophic cardiomyopathy^(7^,^8^,^12^,^13^,^14)^. Alcohol consumption, peripartum cardiomyopathy, and anthracycline exposure may trigger titin-related ACM, which may also be associated with reverse ventricular remodeling with optimal therapy, conduction disorders, and early-onset atrial fibrillation^(6^,^7^,^8^,^13^,^14)^. LMNA-related ACM may exhibit familial partial lipodystrophy, neuromuscular syndromes, a high risk of sudden death, early-onset atrial fibrillation, left bundle branch block (LBBB), atrioventricular block, and a high density of ventricular arrhythmias^(7^,^12^,^13^,^14)^.

Despite considerable progress in understanding the pathophysiology, identification of genetic substrates, and phenotypic characterization of ACM, the boundary between the left-dominant phenotype and DCM is still not well defined in the literature. Some patients with DCM carry genetic variants without, however, showing typical phenotypic features of ACM, such as palpitations, syncope, low QRS voltages in limb leads, inferolateral T wave inversion, and high arrhythmogenic burden. Therefore, it is still unclear whether both phenotypes represent distinct diseases or different presentations within the same pathophysiological spectrum^(6)^.

**Figure 1:**
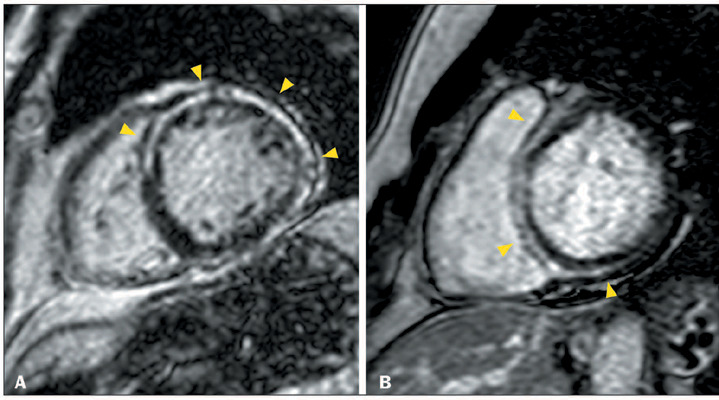
Ring-like LGE in patients with ACM and the hypokinetic nondilated LV phenotype. **A:** CMR showing normal LV volume and systolic function, together with linear ring-like LGE in the mid-anteroseptal, anterior, anterolateral and inferolateral segments (arrowheads), in a 62-year-old man with plakophilin 2-related ACM who presented with palpitations, a family history of ACM inverted T waves and epsilon waves in right precordial leads, and > 500 extrasystoles with LBBB morphology on 24-h Holter monitoring. **B:** CMR showing normal LV volume and systolic function, together with linear ring-like LGE in the basal and mid-anteroseptal, inferoseptal, and inferior segments (arrowheads), in a 46-year-old woman with LMNA-related ACM who had a family history of ACM and aborted sudden death and who presented with first degree atrioventricular block and LBBB.

**Figure 2:**
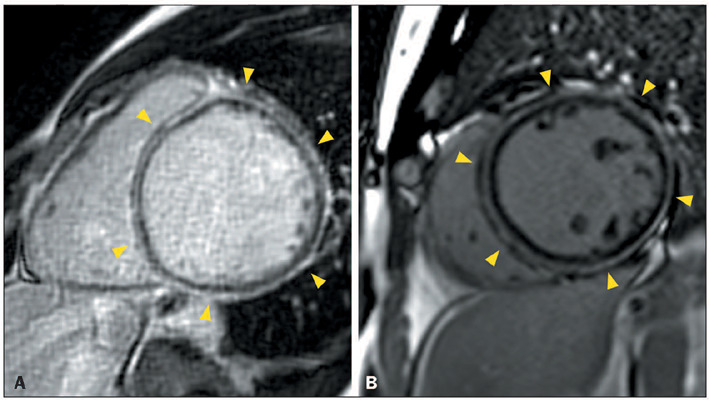
Ring-like LGE in patients with ACM and the dilated LV phenotype. **A:** CMR showing severe LV dilatation and severe systolic dysfunction, together with linear circumferential ring-like LGE in the basal, middle, and apical LV (arrowheads), in a 28-year-old woman with desmoplakin-related ACM who presented with palpitations, heart failure, and a family history of premature sudden death, as well as low QRS voltages in limb leads, epsilon-like waves in inferior and left leads, and inverted T waves in left precordial leads. **B:** CMR showing LV dilatation and systolic dysfunction, together with linear circumferential ringlike LGE in the basal, middle, and apical LV (arrowheads), in a 42-year-old man with filamin-C–related ACM who presented with ventricular arrhythmias and family history of ACM.

**Cardiac imaging** – The predominant ACM phenotype is hypokinetic nondilated LV cardiomyopathy in the early stages (Figure 1). As the disease progresses, dilation results in a DCM phenotype^(6)^, as illustrated in Figure 2. Cine CMR reveals regional wall motion abnormality, global systolic dysfunction, and, in some cases, global dilatation of the LV, RV, or both^(6^,^9^,^12)^. Subepicardial fatty infiltration, not included in the diagnostic criteria, has been observed, sometimes exhibiting a “rat-bite” appearance in long-axis planes, designated the rat-bite sign^(16)^. Although myocardial edema is usually absent, desmoplakin-related ACM may present with “hot-phases”. In these cases, myocardial edema may be detected as hyperintensity on black-blood T2-weighted short-tau inversion-recovery sequences or triple inversion-recovery T2-weighted images (T2WI) or as prolongation of myocardial native T1 and T2 relaxation times^(7^,^12^,^13^,^14)^. LGE detects nonischemic enhancement involving the subepicardial layer, mid-wall layer, or both^(6^,^9^,^12)^.

In a genotype-imaging phenotype study, Augusto et al.^(2)^ demonstrated that ring-like LGE is significantly associated with desmoplakin and filamin-C gene variations. This pattern was also reported in ACM with variations in desmosomal and LMNA genes^(17)^. Ring-like LGE has also been linked to an increased risk of sustained ventricular tachyarrhythmias in nonischemic DCM and ACM^(3^,^5^,^18)^. In a study of 38 cases of ring-like LGE, Bietenbeck et al.^(17)^ identified ACM-related variants in 10 (26%), filamin-C variants in 8 (21%), and LMNA variants in 3 (8%). In the patients with ACM-related variants, the ring-like pattern was more extensive, was more subepicardial, and predominantly involved the free wall. Conversely, in the patients with filamin-C or LMNA variants, the ring-like LGE was more circumferential and predominantly involved the mid-wall.

### Idiopathic DCM

**Definition** – Originally, DCM was defined as ventricular dilation and systolic dysfunction in the absence of systemic arterial hypertension, valvular, congenital or ischemic heart disease^(19)^. However, roughly 50% of individuals who meet those criteria have some underlying condition, including a history of myocarditis, multisystem pathology (autoimmunity, anemia, iron overload, etc.), endocrine disorder (Cushing’s disease, hypothyroidism, hyperthyroidism, or pheochromocytoma), nutritional deficiency (of selenium, zinc, thiamine, etc.), or exposure to toxins such as anthracyclines, 5-fluorouracil, alcohol, amphetamines, cannabis, and cocaine^(20)^. Therefore, the term “idiopathic DCM” has been employed to define cases with no apparent cause. In addition, approximately 25% of such cases have a genetic etiology and are currently referred to as “familial DCM” when at least two closely related family members (first or second degree relatives) meet the diagnostic criteria for idiopathic DCM^(8^,^20)^.

**Etiology** – Familial DCM is usually associated with gene variants of sarcomeric and cytoskeletal proteins, many of which are also associated with ACM^(8^,^20)^. The sarcomeric proteins most commonly affected are titin, betamyosin heavy chain, troponin T2, and alpha-tropomyosin, whereas the cytoskeletal genes most commonly affected are desmin, dystrophin, and filamin-C. Other variants can affect the proteins LMNA, voltage gated sodium channel 5A, tafazzin, RNA binding motif protein 20, and phospholamban^(8^,^20^,^21)^.

**Epidemiology** – The reported prevalence of idiopathic DCM is 7.0–36.5/100,000 population^(19)^.

**Clinical manifestations** – The clinical presentation of idiopathic DCM is marked by dyspnea, lower limb edema, fatigue, and chest pain. Some patients develop atypical chest pain, palpitation, acute decompensation, or cardiogenic shock^(20)^. As previously discussed, there may be overlapping genotypic and phenotypic features between DCM and ACM^(6)^. However, whereas syncope and arrhythmias predominate in ACM, DCM is marked by heart failure and is often associated with LBBB^(8^,^20)^. Therefore, 24-h Holter monitoring is essential to estimate the arrhythmogenic burden, frequent ventricular arrhythmias being more indicative of ACM^(6)^.

**Figure 3:**
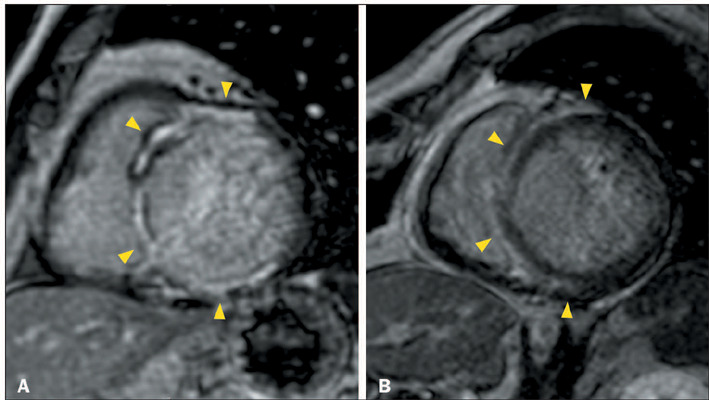
Ring-like LGE in patients with familial DCM. **A:** CMR showing LV dilatation and systolic dysfunction, together with ring-like LGE in the basal and mid-anterior, anteroseptal, inferoseptal, and inferolateral segments (arrowheads), in a 47-year-old man with a troponin T2-related variant who presented with heart failure and a family history of premature sudden death, as well as atrial fibrillation, LBBB, and QRS fragmentation. **B:** CMR showing LV dilatation, systolic dysfunction, normal global native myocardial T1, and minimally increased myocardial extracellular volume, as well as ring-like LGE in the basal and mid-anterior, anteroseptal, inferoseptal, and inferior segments (arrowheads), in a 66-year-old man with a transthyretin-related variant who presented with heart failure, palpitations, and a family history of premature sudden death, together with negative ^99m^Tc-pyrophosphate scintigraphy, first degree atrioventricular block, LBBB, and rare polymorphic ventricular contractions.

**Cardiac imaging** – Cine CMR identifies ventricular dilatation, defined as chamber diameter or volume > 2 standard deviations according to nomograms corrected for age, sex, and body surface area, as well as identifying systolic dysfunction, defined as an ejection fraction < 50%^(20)^, as shown in Figure 3. LGE sequences are essential to rule out ischemic etiology and, in up to one third of DCM cases, may detect linear mid-wall septal fibrosis^(21)^. In addition, native T1 mapping and extracellular volume fraction may be employed to detect diffuse fibrosis and stratify major adverse cardiac events risk in LGE-negative cases^(22^,^23)^. A finding of myocardial edema suggests an inflammatory substrate and an underlying cause for the dilation^(20)^. As previously stated, ring-like LGE was initially described in cardiomyopathies with desmoplakin and filamin-C variants presenting the “arrhythmogenic DCM” or left-dominant ACM phenotypes^(2)^, as depicted in Figure 3. Although the exact prevalence of this enhancement pattern is unknown, its identification is known to be an independent predictor of malignant arrhythmic events^(3^,^18)^.

## INFLAMMATORY CAUSES OF RING-LIKE LGE

Inflammatory causes of ring-like LGE are associated with detection of myocardial edema in CMR. Such causes include acute myocarditis, heart transplant rejection, and cardiac sarcoidosis.

### Acute myocarditis

**Definition** – Myocarditis is an inflammatory disease of the myocardium characterized by an inflammatory cell infiltrate, either with myocyte necrosis (acute myocarditis) or without it (borderline myocarditis). The inflammatory cell type classifies myocarditis into the following varieties^(24^,^25)^: lymphocytic, seen in 55% of individuals submitted to endomyocardial biopsy; borderline, seen in 22%; granulomatous, seen in 10%; eosinophilic, seen in 6%; and giant cell, seen in 6%.

**Etiology** – Lymphocytic myocarditis is usually linked to viral infection, due to direct injury or a post-infectious autoimmune response^(25)^. The most relevant viruses are parvovirus B-19 and human herpesvirus 6, followed by Epstein-Barr virus, an enteroviruses (e.g., Coxsackie B virus), cytomegalovirus, and adenovirus^(26)^.

Eosinophilic myocarditis is associated with hyper-sensitivity (especially to clozapine, carbamazepine, minocycline, β-lactam antibiotics, antitubercular agents, and, less commonly, vaccinations), autoimmunity, eosinophilic granulomatosis with polyangiitis, hypereosinophilic syndromes, parasitic infection (oral transmission of *Toxocara canis*), and, more rarely, paraneoplastic syndromes related to lung cancer^(27)^.

Giant cell myocarditis is attributed to interferon-gamma-induced T-lymphocyte–mediated inflammation^(28)^. Although it occurs primarily in healthy individuals, up to 20% of patients may have other autoimmune diseases^(28)^.

**Epidemiology** – Acute myocarditis predominantly affects young men, with an estimated annual incidence of 1.8 million cases^(25^,^26)^. Eosinophilic and giant cell myocarditis are rare and have an estimated prevalence of up to 0.13/100,000 population^(28)^.

**Clinical manifestations** – The spectrum of clinical presentations of myocarditis includes acute myocarditis—pauci-symptomatic to fulminant, with symptom onset less than one month prior); chronic inflammatory cardiomyopathy—dilated or hypokinetic nondilated phenotypes, with symptoms lasting more than one month; and chronic restrictive cardiomyopathy—secondary to eosinophilic myocarditis and better known as endomyocardial fibrosis^(25^,^27^,^28)^.

Acute myocarditis manifests as flu-like or gastrointestinal prodromes in 18–80% of cases, chest pain in 95%, and dyspnea in 49%, as well as other, nonspecific symptoms such as fever, fatigue, palpitations, and syncope^(26)^. Fulminant myocarditis, rapidly progressive heart failure, ventricular arrhythmias, and atrioventricular block unresponsive to usual therapy within 1–2 weeks should raise the suspicion of eosinophilic or giant cell myocarditis^(25^,^28)^.

**Figure 4:**
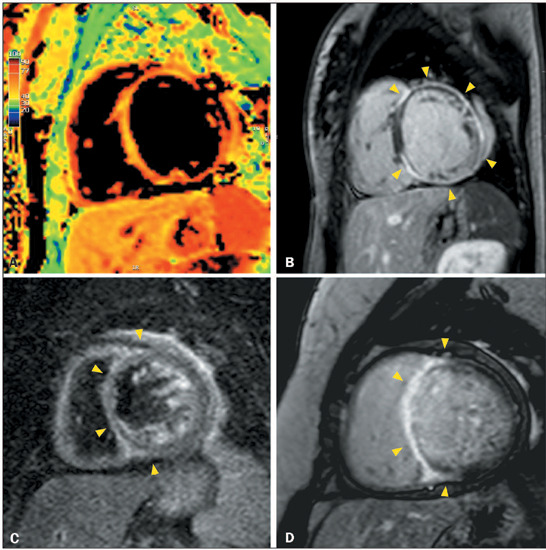
Ring-like LGE in patients with acute myocarditis. **A,B:** CMR showing LV dilatation, systolic dysfunction, and myocardial edema (**A**), with a prolonged T2 relaxation time (67 ms), as well as circumferential ring-like LGE in the middle and apical segments of the LV (arrowheads in **B**), in a 21-year-old woman with biopsy-proven acute lymphocytic myocarditis (presumably with a post-viral etiology) who presented with flu-like prodromes and fulminant myocarditis, with no family history of ACM or sudden premature death. **C,D:** CMR showing LV dilatation, systolic dysfunction, and myocardial edema with T2WI hyperintensity (**C**), together with ring-like LGE in the anterior, anteroseptal, inferoseptal, inferior basal, and inferior middle segments (arrowheads in **D**), in a 52-year-old woman with biopsy proven giant cell myocarditis who presented with fulminant myocarditis and no family history of ACM or sudden premature death.

**Cardiac imaging** – In acute myocarditis, CMR may depict mild ventricular dysfunction, myocardial edema, and tissue necrosis^(29)^. The ejection fraction is preserved in most cases, with only mild focal wall motion abnormalities^(30)^. Myocardial edema is common and manifests as hyperintensity on T2WI sequences or as prolongation of myocardial native T1 and T2 relaxation times^(30)^. Myocardial T2 maps have significantly higher accuracy in detecting acute inflammation than do T2WI and native T1 mapping sequences^(30)^. Myocardial necrosis and the subsequent fibrosis are detected on LGE sequences as subepicardial patchy areas of enhancement, predominantly in the middle and basal inferolateral segments of the LV^(30)^. Subendocardial LGE warrants consideration of giant cell and eosinophilic myocarditis^(27^,^31^,^32)^. In addition, giant cell myocarditis and sarcoidosis may have overlapping findings, with RV and septal wall involvement^(32)^. Up to 9% of patients hospitalized for acute myocarditis develop a fulminant presentation with cardiogenic shock, associated with global systolic dysfunction and extensive LGE, which may take on a ring-like pattern^(3^,^33)^, as illustrated in Figure 4.

### Heart transplant rejection

**Definition** – Cardiac allograft rejection is defined as a host inflammatory response to the transplanted organ. It is classified as hyperacute or acute, with the latter further subdivided into cellular and antibody-mediated rejection^(34^,^35)^.

**Etiology** – Hyperacute allograft rejection is associated with preformed antibodies targeting donor vascular endothelium antigens, such as human leucocyte antigen (HLA) and the ABO system^(34)^. Owing to ABO compatibility testing and panels for reactive antibodies against HLA, hyperacute rejection has become rare^(34)^. Although hyperacute rejection is now well controlled, acute rejection continues to pose a significant challenge during the first year post-transplantation^(34)^. Acute cellular rejection, a T-cell mediated response, leads to myocardial infiltration by lymphocytes and macrophages^(34^,^35)^. In contrast, acute antibody-mediated rejection, triggered by complement activation, results in myocardial arteriolar vasculitis^(34^,^35)^.

**Epidemiology** – Approximately 60% of transplant recipients experience rejection within the first year, with the incidence being highest between 2 and 12 post-procedure^(34^,^36)^. Key risk factors for rejection include young age, Black race, female sex, a greater number of HLA mismatches, high levels of pre-transplant reactive antibodies, a positive donor-specific crossmatch, previous sensitization to OKT3, cytomegalovirus seropositivity, prior ventricular assist device implantation, and retransplantation^(34^,^35)^.

**Clinical manifestations** – Because of the denervation of the transplanted heart, acute transplant rejection is typically asymptomatic or presents with nonspecific and insidious symptoms, such as fatigue, malaise, and dyspnea^(35)^. As rejection progresses, the risk of cardiac allograft vasculopathy and graft failure increases^(34^,^35^,^36)^. Although rare, fulminant rejection can lead to hemodynamic compromise and death^(37^,^38)^. Consequently, regular monitoring is crucial for early diagnosis. Endomyocardial biopsy continues to be the standard method for diagnosing rejection, despite its potential for sampling error and significant interobserver variability^(39)^.

**Figure 5:**
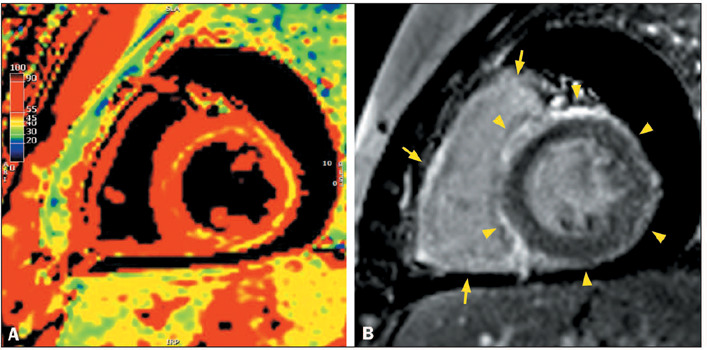
CMR revealed pericardial effusion, normal LV volume and systolic function, myocardial edema (**A**) with prolonged myocardial T2 (58 ms), LGE in the RV (arrows in **B**), and ring-like LGE in the LV (arrowheads in **B**), in a 16-year-old woman who underwent heart transplantation because of idiopathic DCM and developed biopsy-proven acute cellular rejection two years after the surgery.

**Cardiac imaging** – In heart transplant rejection, the primary CMR findings include myocardial thickening, increased LV myocardial mass, myocardial edema, and LGE, with the edema and LGE being more pronounced in the interventricular septum^(34)^. Parametric myocardial mapping techniques reveal increased myocardial native T1 and T2 relaxation times, together with an increased extracellular volume fraction^(36)^. Pericardial effusion, LV size, and ejection fraction lack sensitivity in detecting rejection and are unsuitable for screening purposes^(34)^. In cases of fulminant acute cellular rejection, extensive LGE may be present, often exhibiting a ring-like pattern^(40)^, as depicted in Figure 5.

### Cardiac sarcoidosis

**Definition** – Sarcoidosis is a multisystemic, nonca-seating granulomatous disease of undefined etiology that primarily affects the lungs (in 70% of cases) and the heart (in 25%), followed by the liver, spleen, skin, eyes, and parotid glands^(41)^.

**Etiology** – Cardiac sarcoidosis is likely caused by an immune response to an unidentified antigenic trigger in genetically predisposed individuals^(41^,^42)^. The pathogenesis of sarcoidosis involves the recruitment and activation of macrophages and lymphocytes by interferon-gamma, which leads to the formation of noncaseating epithelioid granulomas and subsequent fibrosis^(42)^.

**Epidemiology** – The prevalence of cardiac sarcoidosis is approximately 5–64/100,000 population, being more common among individuals of African, Scandinavian, or Japanese descent^(41^,^42)^. The disease also exhibits a bimodal age distribution, with peaks at 20 and 50 years of age^(41^,^42)^.

**Clinical manifestations** – Cardiac sarcoidosis reportedly occurs either in isolation (in 5–66% of cases) or together with signs or symptoms of sarcoidosis affecting other organs^(42^,^43)^. The primary manifestations of cardiac sarcoidosis include second or third-degree atrioventricular block, ventricular arrhythmias, syncope, and unexplained nonischemic heart failure in young adults^(41^,^44)^.

**Cardiac imaging** – In its early stages, cardiac sarcoidosis may present as a hypokinetic nondilated LV cardiomyopathy with myocardial inflammatory activity. In some cases, it evolves to a dilated phenotype with myocardial fibrosis and varying degrees of edema. Inflammatory activity can be detected by fluorodeoxyglucose positron-emission tomography (FDG-PET) or CMR^(41)^. On FDG-PET, inflammation is characterized as increased metabolism and glucose uptake^(41)^. On CMR, myocardial inflammation presents as myocardial edema, thickening, and hypokinesia^(41^,^44)^.

In the chronic phase of cardiac sarcoidosis, features such as myocardial thinning, segmental defects, systolic dysfunction, and fibrosis may become apparent^(44)^. LGE typically exhibits a nonischemic pattern, affecting the following layers: subepicardial (in 83% of cases), transmural (in 59%), subendocardial (in 47%), and mid-wall (in 35%). LGE is most commonly observed in the septal region of the LV wall (in 64% of cases), followed by the anterior, lateral, and inferior walls (in 49%, 46%, and 45%, respectively). Along the length of the LV, LGE is most often found in the basal segment (in 59% of cases), followed by the middle segment, in 57%, and the apical segment, in 25%^(41^,^45)^.

**Figure 6:**
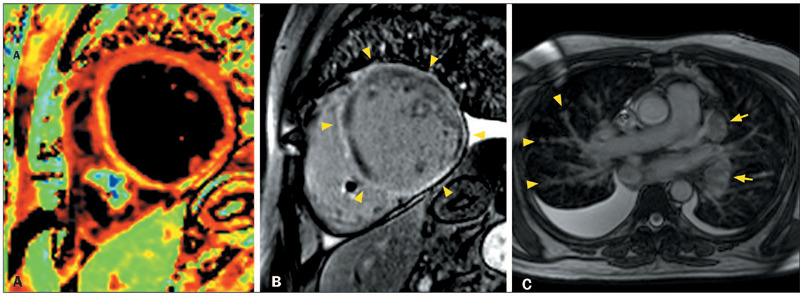
CMR, performed after the implantation of a pacemaker, showing severe LV dilatation, systolic dysfunction, and myocardial edema (**A**), with a prolonged T2 relaxation time (72 ms), together with circumferential ring-like LGE in the basal and middle segments (arrowheads in **B**), in a 48-year-old man with a biopsy-proven cardiac involvement in sarcoidosis who presented with total atrioventricular block. Axial magnetic resonance imaging scan of the chest, showing peribronchovascular interface irregularity (arrowheads in **C**), mediastinal lymph node enlargement (arrows in **C**), and pleural effusion.

When multifocal LGE is present and alternative diagnoses have been excluded, cardiac sarcoidosis is highly probable, especially if there is involvement of the basal septum extending into the RV (hook sign) or significantly extensive LGE^(46)^. Atypical cases may resemble DCM, hypertrophic cardiomyopathy, ACM, ischemic heart disease, or circumferential myocardial fibrosis with a ring-like pattern^(44)^, as shown in Figure 6.

## RING-LIKE MIMICS OF LGE

Although ring-like LGE has most commonly been described in genetic diseases, it can also be encountered in inflammatory cardiomyopathies, albeit a rare finding in the latter. The original description of the ring-like LGE pattern specifically described it as linear, continuous enhancement in the mid-wall or subepicardial layer of at least three adjacent segments^(2)^. Several other cardiomyopathies associated with progressive myocardial fibrosis may mimic the ringlike pattern in their late stages owing to the confluence of multiple foci of enhancement, resulting in circumferential or semi-circumferential LGE. We argue that the irregular appearance, simultaneous involvement of different layers (e.g., foci of subendocardial or transmural extension), and points of discontinuity are essential features to differentiate LGE from these mimics, which may present hypertrophic or dilated phenotypes and will be briefly discussed below, with additional clinical and imaging features shown in Table 2.

**Table 2:** Summary of the clinical, imaging, and ancillary findings of ring-like LGE mimics.

Diagnosis	Clinical context	Electrocardiogram	Magnetic resonance imaging	Ancillary tests
Hypertrophic cardiomyopathy	Dyspnea, syncope, chest pain, arrhythmias, and sudden death	LV overload	Asymmetrical septal LV hypertrophy and ill-defined LGE	Anterior systolic motion of the mitral valve Diastolic dysfunction “Burned out” phase
Danon disease	Skeletal myopathy, learning disabilities, and retinopathy	Atrial and ventricular arrhythmias Pre-excitation	Symmetrical LV hypertrophy Extensive LGE sparing the septum	Increased transaminases, creatine kinase, and troponin
Dystrophin-deficient cardiomyopathy	Muscle weakness Progressive decline in cardiopulmonary capacity	Increased R-S ratio in right precordial leads Deep Q waves in left precordial leads Conduction abnormalities Supraventricular arrhythmias	Inferolateral LGE Fatty replacement of the chest wall muscles	Increased creatine kinase and liver transaminases
Chronic Chagas disease cardiomyopathy	Latin America Arrhythmias, thromboembolism, and heart failure	Bradycardia Right bundle branch block Left anterosuperior fascicular block	“Finger glove” apical aneurysm Inferolateral LGE	Positive *Trypanosoma cruz*i serology
Keshan disease	China Severe malabsorption syndrome Heart failure	Low voltages in limb leads Right bundle-branch block Ventricular or supraventricular arrhythmias Atrioventricular block ST–T segment and T wave abnormalities	Dilatation of the LV Mid-wall LGE	Selenium deficiency Reduced glutathione peroxidase activity

### Hypertrophic cardiomyopathy

**Figure 7:**
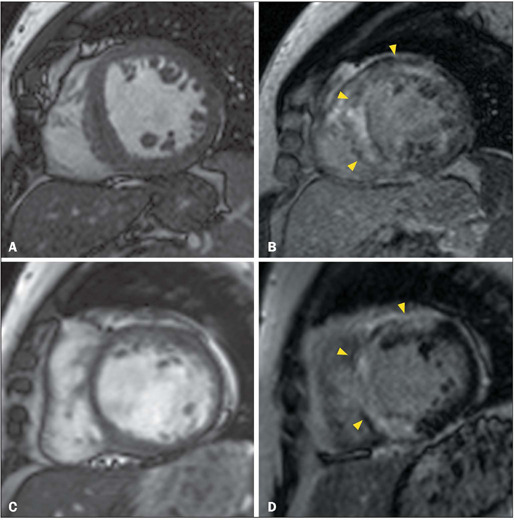
CMR showing non-obstructive septal hypertrophy (**A**), as well as extensive, ill-defined, fibrosis centered in the anterior and septal segments (arrowheads in **B**), in a 54-year-old female with hyper-trophic cardiomyopathy who presented with syncope and progressive dyspnea, together with a family history of sudden death and myocardial hypertrophy. Follow-up CMR, performed eight years later, showing myocardial thinning, ventricular dilatation (**C**), and extensive confluent semi-circumferential myocardial fibrosis (arrowheads in **D**).

Hereditary cardiomyopathy is characterized by LV myocardial hypertrophy and is caused mainly by sarcomeric gene variants^(47^,^48)^. The most common phenotype is asymmetric septal hypertrophy (at least one segment > 15 mm thick), often accompanied by dynamic obstruction of the LV outflow tract, anterior motion of the anterior leaflet of the mitral valve, some degree of diastolic dysfunction, and fibrosis in the interventricular septum and junctional areas. In the later stages (the “burned out” phase), there is a reduction in myocardial thickness, ventricular dilatation, and confluence of enhancement foci that can simulate a DCM phenotype with a ring-like LGE pattern centered on the septal wall (Figure 7).

### Danon disease

**Figure 8:**
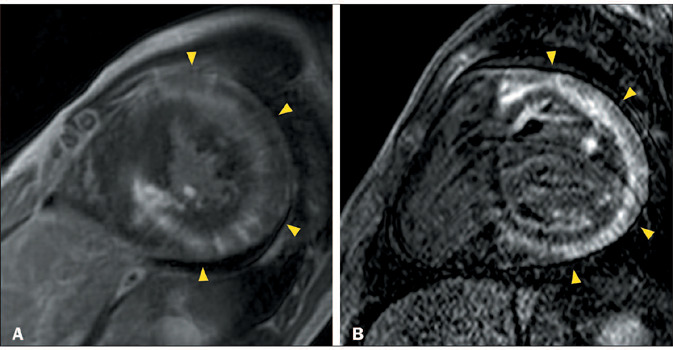
CMR showing non-obstructive, severe, symmetrical LV myocardial hypertrophy, and mid-wall multifocal ill-defined LGE (arrowheads in **A**), with sparing of the middle and basal septal segments, in a 27-year-old man with a previous diagnosis of Danon disease who had a family history of premature sudden death and myocardial hypertrophy. Laboratory tests showed elevated levels of transaminases, creatine kinase, and troponin. ECG revealed PR segment shortening, ventricular extrasystoles, and LV overload. Follow-up magnetic resonance imaging scan, acquired three years later, showing a reduction in myocardial thickness and LV dilation, together with confluent semi-circumferential mid-wall or subepicardial LGE involving in the anterior, anterolateral, infer-olateral, inferior middle, and inferior apical segments (arrowheads in **B**).

Danon disease is a rare X-linked dominant glycogen storage cardiomyopathy related to deficiency of the LAMP2 protein^(49^,^50^,^51)^. The predominant phenotype is severe, symmetrical LV hypertrophy, with extensive lateral and apical myocardial fibrosis. As with hypertrophic cardiomyopathy, Danon disease may also progress to a “burned out” phase in which the fibrosis may take on a ring-like appearance^(49^,^50^,^51)^. However, in these cases, unlike in hypertrophic cardiomyopathy, the fibrosis is centered on the lateral wall (Figure 8).

### Dystrophin-deficient cardiomyopathy

**Figure 9:**
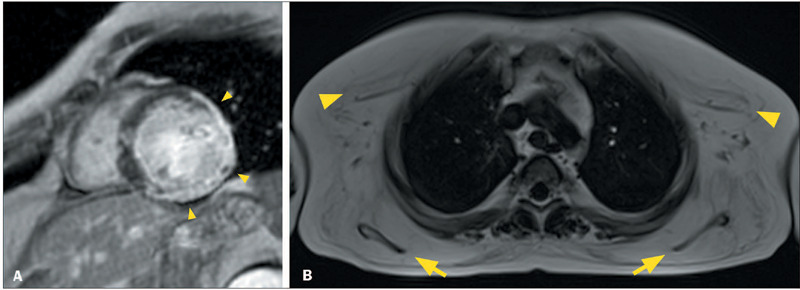
CMR showing severe LV systolic dysfunction and akinesia, as well as extensive semi-circumferential subepicardial LGE in the anterolateral, inferolateral, and inferior segments (arrowheads in **A**), with some transmural extension into the inferolateral segments, in a 22-year-old man with Duchenne muscular dystrophy who presented with progressive muscle weakness, significant physical limitation, and wheelchair dependence. **B:** Magnetic resonance imaging scan of the chest, showing fatty replacement of the muscles in the chest wall, particularly the periscapular muscles (arrows) and pectoralis muscles (arrowheads).

Dystrophin-deficient cardiomyopathy is defined as autosomal recessive X-linked neuromuscular disease that results in the absence or reduced function of dystrophin in Duchenne and Becker muscular dystrophy, respectively^(52^,^53)^. In its early stages, dystrophin-deficient cardiomyopathy may present as a hypokinetic nondilated LV phenotype, with myocardial fibrosis in the subepicardial layer of the inferolateral segments^(54^,^55^,^56)^. As the myocardial fibrosis progresses, systolic dysfunction and LV dilatation occur, and LGE sequences may depict transmural or even circumferential enhancement, mimicking the ring-like pattern^(54^,^55^,^56)^, as illustrated in Figure 9.

### Chronic Chagas disease cardiomyopathy

**Figure 10:**
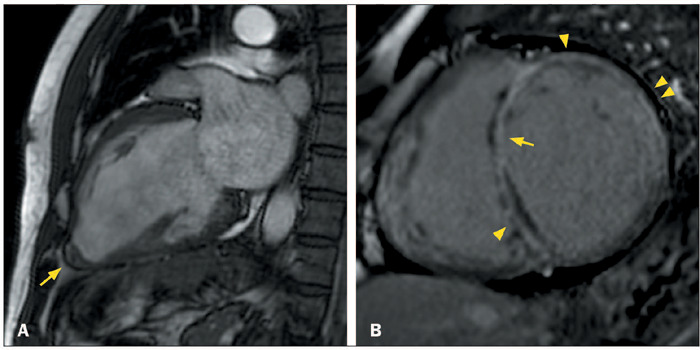
CMR showing LV dilatation, systolic dysfunction, apical aneurysm (arrowhead in **A**), and extensive confluent circumferential LGE (**B**), with a predominant subepicardial component (arrowheads), together with minor subendocardial foci (arrow) and minor transmural foci (double arrowheads), in a 53-year-old man with Chagas disease who presented with heart failure, right bundle block, and left anterosuperior fascicular block.

Chronic Chagas disease cardiomyopathy develops from persistent tissue infection by the protozoan parasite *Trypanosoma cruzi*, resulting in progressive myocardial fibrosis^(57^,^58)^. In its early stages, chronic Chagas disease cardiomyopathy presents as a hypokinetic nondilated LV phenotype, with myocardial fibrosis in the subepicardial layer of the inferolateral segments and apical aneurysms, which are characteristic of Chagas disease, especially when resembling a glove finger^(59^,^60^,^61)^. As the disease progresses, LV dilation and dysfunction are established and LGE progresses, assuming a semi-circumferential distribution, often with transmural extension in the inferolateral segments^(59^,^60^,^61)^, as depicted in Figure 10.

### Keshan disease

**Figure 11:**
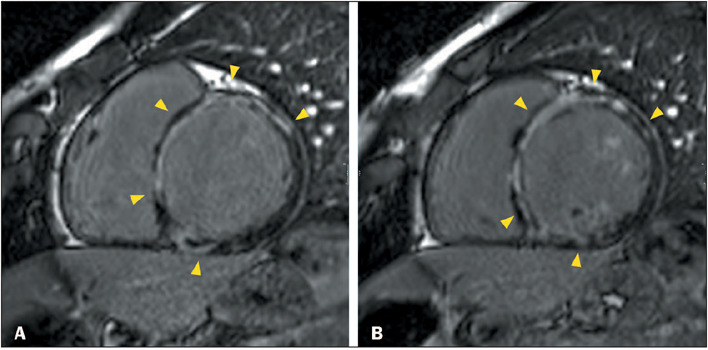
CMR showing LV dilatation and systolic dysfunction, together with extensive, multifocal, irregular, confluent semi-circumferential mid-wall LGE (arrowheads in **A** and **B**), in a 46-year-old man with Keshan disease who had previously undergone gastroplasty and presented with a severe malabsorption syndrome and subsequent heart failure. Laboratory tests revealed severe selenium deficiency.

Keshan disease is a dilated cardiomyopathy that is endemic in Keshan County, western Heilongjiang province, China, caused by selenium deficiency or related to severe malabsorption syndromes^(62^,^63^,^64)^. CMR can reveal DCM with progressive myocardial mid-wall fibrosis that, in some cases, assumes a circumferential distribution mimicking the ring-like pattern^(63^,^65^,^66)^, as shown in Figure 11.

## CONCLUSION

The ring-like LGE pattern was initially described in genetic cardiomyopathies with arrhythmogenic and dilated phenotypes. However, this pattern can also be seen in inflammatory cardiomyopathies, although it is not a common finding. In addition, other cardiomyopathies with progressive fibrosis may simulate the ring-like pattern in the late stages due to confluence of fibrotic foci. Integrating additional imaging features, electrocardiographic findings, and clinical parameters improves the ability to establish a comprehensive differential diagnosis among the various underlying causes of ring-like LGE and its mimics.
